# Weak Intermolecular Interactions in a Series of Bioactive Oxazoles

**DOI:** 10.3390/molecules26103024

**Published:** 2021-05-19

**Authors:** Anita M. Grześkiewicz, Tomasz Stefański, Maciej Kubicki

**Affiliations:** Faculty of Chemistry, Adam Mickiewicz University, Uniwersytetu Poznanskiego 8, 61-614 Poznań, Poland; aniow@amu.edu.pl (A.M.G.); tomasz.stefanski@amu.edu.pl (T.S.)

**Keywords:** oxazoles, intermolecular interactions, topological analysis

## Abstract

The intermolecular interactions in a series of nine similar 4,5-phenyl-oxazoles were studied on the basis of crystal structures determined by X-ray diffraction. The crystal architectures were analyzed for the importance and hierarchies of different, weak intermolecular interactions using three approaches: the geometrical characteristics, topological analysis (for the model based on the transfer of multipolar parameters), and energetics of the molecule–molecule interactions. The geometries of the molecules were quite similar and close to the typical values. The results of the analysis of the interactions suggest that the number of nonspecific interactions is more important than the apparent strength of the specific interactions. The interactions involving covalently bound bromine and divalent sulfur atoms were classified as secondary, they certainly did not define the crystal packing, and they played a minor role in the overall crystal cohesion energies. Incidentally, another method for confirming the degree of isostructurality, according to the topologies of the interactions, is described.

## 1. Introduction

Intermolecular interactions (specific, such as hydrogen bonds, or nonspecific, such as van der Waals interactions) constitute principal factors in molecular recognition and, as a consequence, biological activity. Therefore, knowledge about the presence, hierarchy, relative energies, and importance of different interactions is crucial in explaining the biological action of given compounds and in designing new and more active or more specific molecules. The situation seems to be especially profitable if there is a series of similar compounds available, when small differences in the molecular structure may be related to significant changes in crystal architectures, i.e., supramolecules par excellence (citing Dunitz’s famous definition [1,2]).

In the course of such research, a plethora of more or less important kinds of specific interactions have been proposed, analyzed, described, and tentatively explained. From the classical hydrogen bonds of, for instance, the O–H···O=C type, through weaker interactions involving hydrogen atoms (hydrogen bridges, using the formula of Desiraju [3]), halogen, chalcogen, pnicogen, and tetrel interactions, or π···π, cation···π, and anion···π interactions, to quite exotic ones, such as hydrogen···hydrogen, there are several scholars dealing with these phenomena in the literature (e.g., [4,5,6,7,8]). 

Meanwhile, Dunitz and Gavezzotti [9] started a relevant discussion on the role and importance of intermolecular specific interactions of the types listed above (with the notable exception of the classical, strong hydrogen bonds) for crystal architecture with respect to the more diffuse, delocalized interactions between the molecular electron density distributions. They posited that “one cannot deny that these weak intermolecular atom–atom bonds can be neatly categorized on the basis of geometrical, spectroscopic, and even energetic criteria (…). The question is not whether weak hydrogen bonds ‘exist’, but rather to what extent are they relevant in distinguishing one possible crystal structure from another?” This discussion has been continued (see, for instance, the exchange in IUCrJ in 2015 [10,11,12]). Another important advance in the understanding of the role of interactions or energies, from different points of view, can be related to the works of Wozniak et al., who identified the continua of atom–atom interactions, from covalent to very weak, almost van der Waals type [13,14], and to the work of Spackman, who showed that the dependence of both kinetic and potential energies on the H···O distance for “weak” hydrogen bonds, determined by Espinosa, Molins, and Lecomte [15] on the basis of multipolar model and high-resolution diffraction data, can be, in principle, obtained from a simple, independent atom model (promolecule) [16]. Further studies, e.g., by Gatti et al. [17], have shown that there are instances when the pro-molecular model yields different topologies, compared to the corresponding multipole or theoretical densities.

Therefore, we decided to compare different viewpoints on intermolecular interactions on the basis of the abovementioned discussion. We use three kinds of descriptions: (1) the geometry of interactions (weak hydrogen bonds, π···π interactions, H···H contacts, and other van der Waals contacts, i.e., generally, contacts with a name); (2) the topological (atoms-in-molecules type [18]) descriptors of these contacts; and (3) the interaction energies between the pairs of molecules, which lead to the packing energies of the crystals. For this last part, two methods with relatively quick calculations are used, in principle, to study the tendencies, rather than individual values: the PIXEL method, included in the Mercury software [19,20,21], and HF-3-21G, included in the CrystalExplorer software [22]. In all three methods, the molecular model obtained by means of standard-resolution X-ray diffraction data were used, with X–H bonds elongated to the typical neutron diffraction values.

**Scheme 1:**
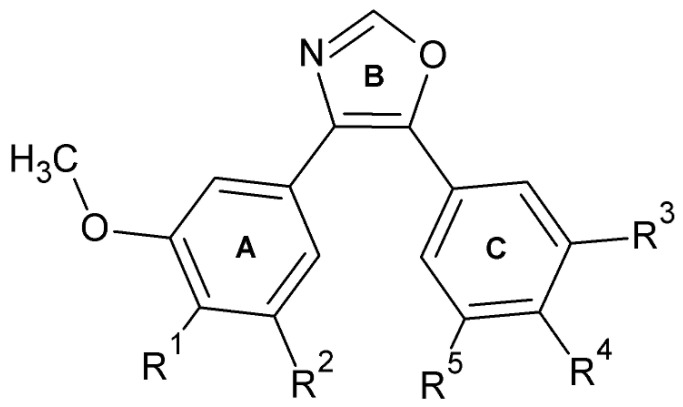
The molecular skeleton together with the ring naming. **1**: R^1^=R^2^=R^3^=OCH_3_, R^4^=SCH_3_, R^5^=H; **2**: R^1^=R^2^=R^3^=OCH_3_, R^4^=SCH_3_, R^5^=Br; **3**: R^1^=R^2^= R^3^=OCH_3_, R^4^=H, R^5^=SH; **4**: R^1^=SCH3, R^2^=R^4^=OCH_3_, R^3^=OH, R^5^=H; **5**: R^1^=SCH_3_, R^2^=R^4^=OCH_3_, R^3^=F, R^5^=H; **6**: R^1^=SCH_3_, R^2^=OCH_3_, R^3^=R^5^=H, R^4^=OC_2_H_5_; **7**: R^1^=R^4^=OCH_3_, R^2^=Br, R^3^=SCH_3_, R^5^=H; **8**: R^1^=R^3^=OCH_3_, R^2^=Br, R^4^=SCH_3_, R^5^=H; **9**: R^1^= SCH_3_, R^2^=Br, R^3^=OH, R^4^=OCH_3_, R^5^=H.

Here, we present the results of this in-depth analysis (geometrical, energetical, and topological) of weak intermolecular interactions in a series of 4,5-diaromatic-substituted oxazoles: **1**: 5-[3-methoxy-4-(methylsulfanyl)phenyl]-4-(3,4,5-trimethoxyphenyl)-1,3-oxazole; **2**: 5-[3-bromo-5-methoxy-4-(methylsulfanyl)phenyl]-4-(3,4,5-trimethoxyphenyl)-1,3-oxazole; **3**: 2-methoxy-5-[4-(3,4,5-trimethoxyphenyl)-1,3-oxazol-5-yl]benzenethiol; **4**: 5-{4-[3,5-dimethoxy-4-(methylsulfanyl)phenyl]-1,3-oxazol-5-yl}-2-methoxyphenol; **5**: 4-[3,5-dimethoxy-4-(methylsulfanyl)phenyl]-5-(3-fluoro-4-methoxyphenyl)-1,3-oxazole; **6**: 4-[3,5-dimethoxy-4-(methylsulfanyl)phenyl]-5-(4-ethoxyphenyl)-1,3-oxazole; **7**: 4-(3-bromo-4,5-dimethoxyphenyl)-5-[4-methoxy-3-(methylsulfanyl)phenyl]-1,3-oxazole; **8**: 4-[3-bromo-5-methoxy-4-(methylsulfanyl)phenyl]-5-[3-methoxy-4-(methylsulfanyl)phenyl]-1,3-oxazole; and **9**: 5-{4-[3-bromo-5-methoxy-4-(methylsulfanyl)phenyl]-1,3-oxazol-5-yl}-2-methoxyphenol (cf. Scheme 1).

These compounds were synthesized as *cis*-restricted analogues of combretastin A-4 (CA-4), a strong inhibitor of tubulin polymerization, with the potentially profitable methylthio substituent in one of the phenyl rings [23]. For instance, compounds **4** and **7** efficiently inhibited tubulin polymerization, with IC_50_ values below 1 μM; moreover, it was shown that this activity was 5-fold higher than that for OMe analogues. The effects on cell cycle distribution and proapoptotic activities of these compounds were comparable to those observed for CA-4 [23].

Besides, all these compounds provide a number of different possibilities relating to intermolecular interactions, while maintaining the main skeleton of the molecules.

## 2. Results

Scheme 1 shows the general structure of compounds **1**–**9**, together with the ring naming. Depending on the substitution pattern in ring A, all molecules were divided into three groups (see Scheme 1): 3,4,5-trimethoxy derivatives (**1**–**3**), 3,5-dimethoxy-4-thiomethoxy derivatives (**4**–**6**), and 3-bromo-4,5-dimethoxy or 3-bromo-4-methoxythio-5-methoxy derivatives (**7**–**9**).

**Figure 1:**
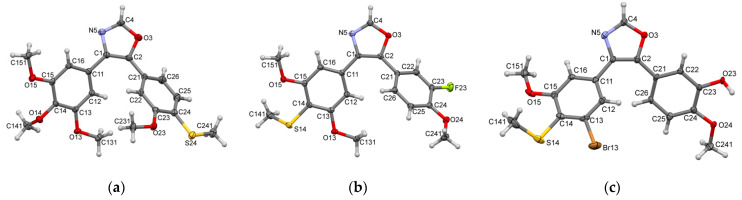
Perspective views for the chosen molecules from each group: (**a**) **1**, (**b**) **5**, (**c**) **9**. Ellipsoids are drawn at the 50% probability level, and hydrogen atoms are shown as spheres of arbitrary radii.

**Figure 2:**
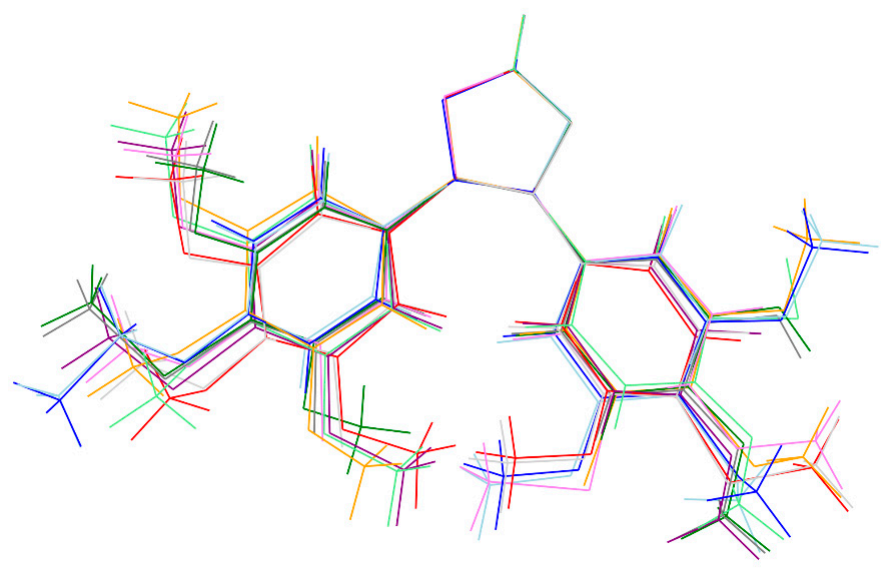
A comparison of all molecules. The oxazole rings were fitted onto one another.

**Figure 3:**
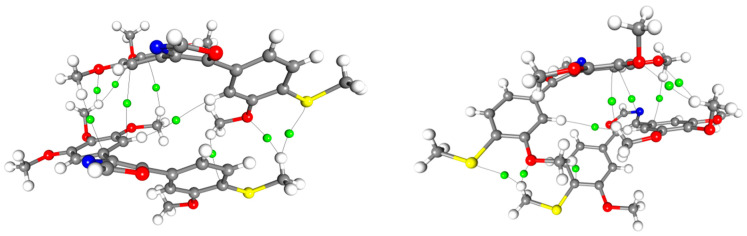
Two of the most important (with the highest interaction energies) motifs in the crystal structure **1** (cf. text). Green circles show the positions of the (3, −1) critical points.

The perspective views of the representative examples from each group are shown in Figure 1, Figure 2 and Figure 3 (the remaining are submitted as Figures S1–S6, Supplementary Materials), and a comparison of some relevant geometrical characteristics for all compounds is given in Table 1. As shown by the values in this table, there were no significant differences in the overall conformations of the molecules, as additionally shown in Figure 2, which presents the result of the overlap of all molecules on the planes of the oxazole ring. Furthermore, the conformation of OMe or SMe substituents was typical (see Table 1), exhibiting a roughly coplanar disposition with respect to the aromatic ring for groups without two neighboring non-hydrogen substituents and an almost perpendicular disposition for the groups with such substituents in both neighboring positions.

The geometry of the oxazole ring was also quite typical, with the characteristic pattern of shorter and longer bonds, generally in agreement with the chemical formula (double C1=C2 and C4=N5 bonds; see Table 1). Very similar values can be found in other structures of molecules containing the neutral oxazole ring. The last column in Table 1 presents the results found in the Cambridge Structural Database ([24]; version 5.42 from November 2020; non-disordered structures only); the upper row presents 1,2-diaromatic-substituted compounds (38 fragments found in the CDB); and the lower row presents all compounds without rings fused to the oxazole one (363 hits).

The similarities of the structures, together with the relatively wide palette of point-like differences, allowed for systematic studies of the subtle pattern of different intermolecular interactions determining the crystal architectures. The apparent lack of “classical” hydrogen-bond donors and acceptors in the majority of compounds makes these series useful for classifying weaker interactions.

According to the theory of atoms in molecules [18], the calculation of the electron density gradient allowed us to locate the critical points (CP), where ∇ρ(**r**) = 0. The nature of the critical point was determined by analyzing the principal axes (eigenvectors) and curvatures (eigenvalues) of the Hessian matrix {*∂*^2^ρ/*∂*x_i_*∂*x_j_}. Each CP was characterized by a (ω, σ) pair, where ω is the number of nonzero eigenvalues, and σ is the sum of their signs (signature). For example, a (3, −1) bond critical point has three nonzero eigenvalues, two of them being negative, and one being positive. Generally, a covalent bond has a (3, −1) CP associated with a large electron density ρ(**r**) and a negative Laplacian ∇^2^ρ(**r**). On the other hand, ionic and hydrogen bonds or van der Waals interactions have a (3, −1) CP associated with a lower ρ(**r**) and a positive Laplacian.

A full analysis of all pairs of molecules, for which the critical points were found, is presented in Tables S1–S8 (Supplementary Materials). Each table contains a list of contacts with geometrical characteristics, topological parameters (electron density and Laplacian values at the critical points), and energies of interactions for the certain pair calculated using the PIXEL and HF methods.

Here, we only analyze some of the most important (with the highest interaction energies) and most interesting interactions between the pairs of molecules.

In the case of **1** (Table 2 lists the relevant data), the two motifs with the highest interaction energies, summing to more than half of the total interaction energy of the structure, together with the positions of the critical points, are shown in Figure 3.

In the first motif, i.e., an infinite chain of molecules along the *x-*direction (related to the unit cell with vector 5.1299 (4) Å), as many as nine (3, −1) critical points between the subsequent molecules were found. The second motif was a centrosymmetric (1 − *x*, 1 − *y*, 1 − *z*) dimer with five pairs of CPs. While the characteristics of all these CPs (density and Laplacian values) were not particularly prominent (in fact, some of them are clearly dubious), altogether, these contacts produced quite an important share of the total interaction energies (−202.3 kJ/mol for PIXEL; −205.0 kJ/mol for HF). In fact, for these pairs, there were hardly any contacts that could be clearly related to a well-defined “interaction”, in the sense of atom···atom pairwise interactions. On the contrary, they seemed to be good examples of more delocalized, overall contacts, contributing an important part of the cohesion energy of the crystals.

The next two pairs were also quite typical and interesting. In these cases, there were better defined “interactions” of the C–H···O (2.34 Å) and C–H···N (2.48 Å) type. These contacts were connected to the best defined critical points, with relatively high electron density values, and, probably more importantly, outstanding Laplacian values. This may be related to the much smaller share of dispersion energy component E_dis_ values for the HF method. These interactions had a much smaller importance for the PIXEL method, which could be related to the relatively small “contact” areas. In this case, we also checked, for the sake of comparison, the tendencies using the DFT method (B3LYP/6-31G(d,p)), and the results were similar: The tendencies were the same, and the values did not differ much (Table 3).

In a few cases, there were classical, medium-strength hydrogen bonds, but the abovementioned features were also preserved in these cases. For instance, in **4** (Table 4), the highest interaction energy was calculated for a pair with as many as 11 critical bond points, with a low or even very low density and Laplacian values. On the other hand, for a pair connected by an O–H···N hydrogen bond, accompanied by a relatively short and linear C–H···O bridge, the energy was lower (comparable for HF; much lower for PIXEL), and the same observation regarding dispersion energy was observed here. The exact same situation was observed in **9** (isostructural pair).

These features were generally observed in all cases. Such a wide comparison of, in principle, similar compounds might be regarded as an important addition to the deeper insight into the nature of intermolecular interactions, as well as their specificity, compared to covalent or generally intramolecular bonds, and the delicate hierarchies of the factors responsible for creating the internal architecture of molecular crystals.

**Figure 4:**
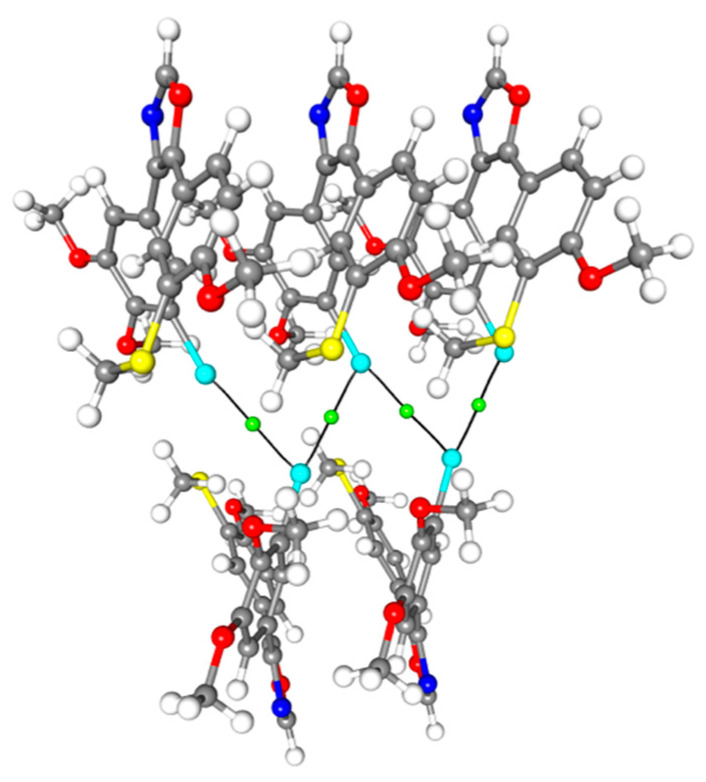
Br···Br motifs in structure **7**, cf. text. Green dots show the positions of critical points.

A bromine atom was only occasionally involved in important interactions. The best example was structure **7** (Table 5 and Figure 4), where one can find (using atom–atom interaction language) Br···Br interactions, fitting quite well into the halogen-bond description (Br···Br 3.564Å, C-Br···Br 165.3° and 120.1°), accompanied by the secondary C–Br···O interaction, with a much more exotic geometry. These interactions took part in the construction of the pair of molecules with the highest interaction energies. Over 10 critical points were found for this pair of molecules. In the other cases, Br was only involved in weak secondary or tertiary C–H···Br contacts, for which the interaction paths were determined, together with the appropriate critical points, but they were highly unspecific.

It is worth noting, in the context of discussing the role of covalently bound Br atoms, that compounds **4** and **9** were highly isostructural (cf. Figure S7, Supplementary Information). The conventional isostructurality indices presented very high values: unit cell similarities [25] 0.006 (ideal value 0), elongation [26] 0.003 (0), and isostructurality index [25] 0.985 (1) (methyl (c131) omitted). Furthermore, a comparison of the interaction data (Supplementary Materials) showed that the energetically most important pairs of molecules were almost exactly the same in both cases. Thus, exchanging the Br with a methyl group did not change the overall picture of the interaction energies, and the interactions with Br were of secondary (at best) importance for the determination of the crystal architecture.

On the other hand, some relatively close similarity could be observed between structures **3** and **5** (similarity 0.013, elongation 0.001, and isostructurality index 0.91). In these cases, however, the details of the interactions (Supplementary Materials) showed only a vague similarity in the pattern of interaction energies; therefore, such an analysis of intermolecular interactions may be regarded as an additional (and, in fact, crucial) method for checking the relevance of crystal structure similarities.

**Figure 5:**
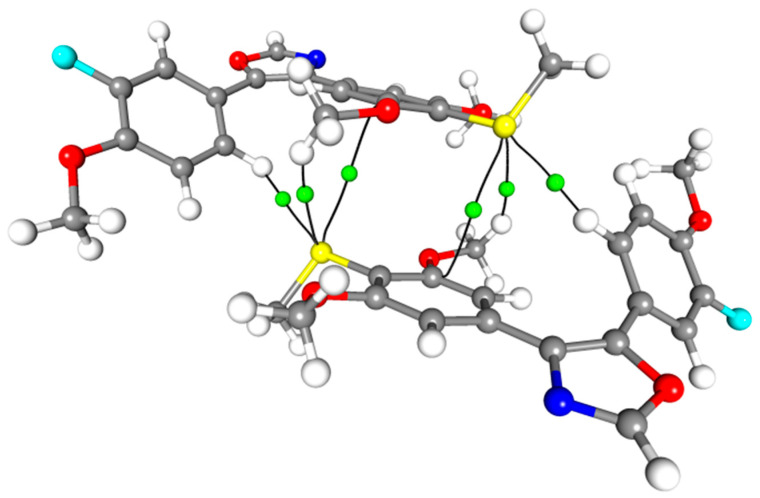
The contacts involving sulphur atoms in structure **5** (cf. Text ). Green circles denote the critical bond points.

Sulfur atoms, although present in almost all molecules, are rarely involved in contacts outside of the geometrically enforced C–H···S or C···S contacts. In the case of **5**, however, there was a centrosymmetric pair of molecules (Figure 5 and Table 6), in all contacts of which sulfur atoms were involved, having critical points (and interaction paths) that added quite a reasonable interaction energy (confirmed by all methods).

## 3. Materials and Methods

The general protocol underlying the synthesis and spectroscopic data for some of the compounds was previously described [23]. Diffraction data were collected using the ω-scan technique for **6** and **8** at 130(1) on a Rigaku SuperNova four-circle diffractometer with an Atlas CCD using detector mirror-monochromated CuK_α_ radiation (λ = 1.54178 Å) and, for all other cases, on a Rigaku XCalibur four-circle diffractometer with an EOS CCD detector and graphite-monochromated MoK_α_ radiation (λ = 0.71073 Å; **1**, **3**, **5**, and **9** at 100(1) K; **2**, **4**, and **7** at room temperature). The data were corrected for Lorentz polarization, as well as for absorption effects [27]. Precise unit-cell parameters were determined by a least-squares fit of the reflections with the highest intensity, chosen from the whole experiment. The structures were solved with SHELXT [28] and refined with the full-matrix least-squares procedure on F^2^ by SHELXL [29]. All non-hydrogen atoms were refined anisotropically. SH (**3**) and OH (**4**) hydrogen atoms were found in the different Fourier maps and freely refined, whereas all the other hydrogen atoms were placed in idealized positions and refined as the ‘riding model’, with isotropic displacement parameters set to 1.2 (1.5 for CH_3_) times the U_eq_ of appropriate carrier atoms. The crystals of **6** turned out to be twinned, and this was considered during both data reduction and structure refinement. The BASF parameter, indicating the mutual content of two components, was refined at 0.199(5). In structures **2** and **8**, weak restraints for the displacement ellipsoids were applied.

Table 7 lists relevant crystallographic data, together with details of the refinement procedure.

**Energy calculations.** The calculations of the interaction energies between pairs of molecules and packing energies were performed using two methods:

(a) Wavefunctions at the HF/6-31G(d,p) level (hereinafter: HF). The energy of the interaction was calculated as follows using the CrystalExplorer software [22] in terms of four key components: electrostatic, polarization, dispersion, and exchange–repulsion:

E_tot_ = k_ele_E_ele_ + k_pol_E_pol_ + k_dis_E_dis_ + kr_ep_E_rep_;

(b) The PIXEL method [23,24], included in the Mercury program [24].

In both cases, the hydrogen atoms were moved to the average geometry, as determined by neutron diffraction.

**AIM topological analysis.** The topology (atoms-in-molecules [18]) of the electron density distribution was calculated using the MoPro software [30]. In our previous studies [31], we checked different models of electron density, and the superiority of the model with multipolar parameters transferred from the ELMAM2 database (experimental databank of transferable multipolar atom models) [31] was shown [32].

As not all atom types were available, we used some approximations in proceeding with the transfer. For instance, in the oxazole ring, multipolar parameters for atoms C2, C4, and H4 were transferred from analogous structures, replacing the oxygen atom with a nitrogen one. A similar approximation was used for C131, C141, C151, C231, and the corresponding atoms.

## 4. Conclusions

The crystal structures of nine closely related, biologically active oxazole derivatives were determined by means of X-ray diffraction, and an in-depth analysis of weak intermolecular interactions was performed. For this, the geometry, topology of the electron density distribution, and the interaction energies were determined, and the relationships among these aspects were analyzed. It is suggested that, even in the presence of medium-strength hydrogen bonds, the more diffused, less specific interactions are generally more important for the cohesion energies. The interactions involving Br or S atoms were generally found to be secondary, and a modification of the analysis of the phenomenon of isostructurality was proposed.

## Figures and Tables

**Table 1:** Relevant geometrical parameters of the studied compounds (Å, °), with the s.u.’s in parentheses. The second line in column **8**, if it exists, refers to the less-occupied alternative. A, B, C denote the planes of the rings, according to Scheme 1, O(S)Mex, etc.—the methoxy or thiometoxy group at position x; A/B, etc., are dihedral angles between appropriate least-squares planes. In the structure of **6**, there are two symmetry-independent molecules, denoted here as **6A** and **6B**.

	1	2	3	4	5	6A	6B	7	8	9	CSDB
C1-C2	1.355(4)	1.350(6)	1.367(2)	1.363(5)	1.3595(19)	1.362(4)	1.363(4)	1.354(11)	1.361(3)	1.354(5)	1.357(14) 1.348(15)
C1-N5	1.403(3)	1.387(6)	1.408(2)	1.409(4)	1.4099(17)	1.401(4)	1.401(4)	1.423(10)	1.397(3)	1.404(5)	1.398(10) 1.393(12)
C2-O3	1.385(3)	1.381(5)	1.3906(18)	1.395(4)	1.3899(16)	1.391(3)	1.391(3)	1.376(9)	1.378(3)	1.387(5)	1.296(15) 1.294(15)
O3-C4	1.352(3)	1.346(5)	1.3504(19)	1.346(4)	1.3550(16)	1.347(4)	1.353(4)	1.350(10)	1.356(4)	1.345(4)	1.350(16) 1.359(18)
C4-N5	1.287(4)	1.256(6)	1.292(2)	1.287(5)	1.2874(18)	1.276(4)	1.275(4)	1.286(11)	1.276(4)	1.288(5)	1.387(11) 1.379(13)
C2-C1-N5	108.8(3)	109.0(4)	108.62(13)	108.5(3)	108.51(12)	108.9(3)	109.0(2)	109.5(8)	109.1(2)	108.8(4)	108.6(6) 109.5(11)
C1-C2-O3	107.8(2)	107.1(5)	107.25(13)	107.0(3)	107.57(12)	106.8(3)	106.8(3)	107.2(8)	107.2(2)	107.1(3)	107.4(6) 107.4(11)
C2-O3-C4	103.9(2)	103.3(4)	104.54(12)	104.9(3)	104.36(10)	104.4(2)	104.2(2)	104.5(7)	104.0(2)	104.9(3)	104.9(8) 104.7(9)
O3-C4-N5	115.3(3)	116.2(5)	115.06(14)	114.8(3)	114.85(12)	115.3(3)	115.4(3)	116.1(9)	115.5(3)	114.5(4)	114.0(11) 113.9(10)
C1-N5-C4	104.3(2)	104.3(4)	104.50(13)	104.9(3)	104.68(11)	104.6(3)	104.5(3)	102.6(8)	104.1(2)	104.7(3)	105.1(8) 104.6(9)
C12-C13-C14	120.5(3)	119.3(5)	120.42(14)	121.3(3)	120.62(13)	121.5(3)	121.3(3)	121.8(8)	121.7(4)	122.9(4)	
C13-C14-C15	119.7(3)	119.2(5)	119.60(14)	118.4(3)	118.46(12)	117.9(2)	118.4(2)	119.9(9)	118.8(2)	116.8(4)	
C14-C15-C16	120.1(3)	120.6(5)	120.28(14)	120.8(3)	121.24(12)	120.8(3)	120.3(3)	116.7(10)	119.4(3)	121.0(4)	
C22-C23-C24	121.1(3)	122.6(5)	119.76(14)	120.2(3)	123.31(13)	119.7(3)	120.0(3)	117.3(9)	120.7(2)	119.6(4)	
C23-C24-C25	118.2(3)	116.2(4)	119.60(14)	119.3(3)	117.34(13)	119.7(3)	119.9(3)	121.5(9)	118.4(2)	120.6(4)	
C2-C1-C11-C12	−26.2(5)	−16.8(9)	−13.0(3)	−40.5(6)	−18.1(3)	−7.1(6)	−20.8(6)	−30.5(15)	−28.6(10) 144.9(13)	−39.6(7)	
C2-C1-C11-C16	154.4(3)	162.5(5)	168.67(16)	142.1(4)	163.19(14)	173.6(4)	161.2(4)	152.9(10)	146.6(6) −54(2)	144.2(5)	
N5-C1-C11-C12	149.3(3)	163.3(5)	163.65(14)	140.2(4)	158.95(13)	171.5(3)	157.7(3)	148.1(8)	143.7(7) −22(2)	141.0(4)	
N5-C1-C11-C16	−30.1(4)	−17.4(7)	−14.6(2)	−37.3(5)	−19.78(19)	−7.8(5)	−20.4(5)	−28.5(13)	−41.1(9) 139.7(13)	−35.2(6)	
C1-C2-C21-C22	−36.1(6)	−28.4(9)	147.51(19)	154.5(4)	143.26(17)	139.7(5)	145.3(4)	−22.2(18)	−30.5(5)	153.8(5)	
C1-C2-C21-C26	142.4(4)	155.0(6)	−38.3(3)	−26.5(7)	−38.2(3)	−40.5(7)	−34.9(6)	162.0(12)	151.6(4)	−28.3(8)	
O3-C2-C21-C22	139.8(3)	149.4(5)	−35.70(19)	−28.2(5)	−39.97(18)	−37.5(5)	−31.5(4)	157.1(8)	145.1(3)	−28.9(6)	
O3-C2-C21-C26	−41.7(4)	−27.2(7)	138.51(14)	150.8(3)	138.57(13)	142.3(3)	148.3(3)	−18.7(13)	−32.9(4)	149.0(4)	
A/B	28.36(14)	17.04(18)	14.51(6)	39.20(10)	19.42(7)	7.3(3)	20.56(19)	30.8(3)	35.4(2)	37.53(11)	
B/C	39.72(14)	27.75(11)	38.28(5)	28.08(16)	39.63(4)	38.46(14)	32.64(18)	20.2(4)	32.79(17)	29.31(18)	
A/C	51.51(11)	32.47(12)	47.42(4)	51.84(11)	47.83(4)	38.69(1)	39.00(14)	42.5(3)	49.6(2)	51.54(13)	
A/OMe3	6.5(2)	4.7(3)	3.15(14)	8.3(2)	13.09(15)	2.0(7)	5.6(5)				
A/O(S)Me4	73.27(13)	81.2(3)	80.09(13)	50.39(13)	60.10(5)	86.7(2)	69.44(15)	79.4(5)	79.6(3)	50.25(16)	
A/OMe5	0.9(2)	6.6(3)	4.09(15)	9.0(3)	4.84(17)	8.7(5)	3.5(5)	2.5(7)	5.6(7)	7.3(3)	
B/OMe3	12.3(3)	3.24(18)						1.3(5)	6.19(18)		
B/SMe4	1.2(2)	85.6(2)							1.2(2)		
B/OMe4			0.80(16)	1.3(5)	4.42(17)	3.10(17)	1.4(5)	2.3(11)		2.3(5)	

**Table 2:** Details of the interactions for the chosen pairs of molecules in **1** (cf. Text). G_cp_: kinetic energy density (kJ/mol/Bohr^3^); V_cp_: potential energy density (kJ/mol/Bohr^3^); Lap: laplacian at the BCP (e·Å^−5^)]; Den: electron density at the BCP (e·Å^−3^), distances in Å, angles in °, and energies in kJ/mol. Symmetry codes: ^i^ −1 + x,y,z; ^ii^ 1 − x,1 − y,1 − z; ^iii^ −x,1 − y,1 − z; ^iv^ 1 − x,2 − y,2 − z.

Atom1	Atom2	Gcp	Vcp	DEN	LAP	X···Y	H···Y	X-H···Y	Pix	HF *B3LYP*
C16	C12 ^i^	4.35	−2.77	0.01834	0.218	3.742			−78.9	−69.1 *−67.4*
C14	H13C ^i^	8.55	−6.18	0.03675	0.401	3.717	2.90	131		
O15	H14B ^i^	10.27	−6.41	0.02888	0.519	3.447	2.78	119		
O14	H14B ^i^	3.86	−2.43	0.01651	0.194	4.173	3.14	158		
H15C	C11 ^i^	8.86	−7.02	0.0442	0.393	3.980	2.90	170		
H22	H12 ^i^	0.98	−0.58	0.00581	0.051	3.39				
O23	H24A ^i^	14.83	−10.65	0.05047	0.698	3.452	2.53	142		
H23C	C24 ^i^	12.94	−10.26	0.05547	0.574	3.550	2.69	136		
S24	H24A ^i^	9.07	−6.53	0.03773	0.427	3.894	2.96	144		
H13A	O13 ^ii^	12.71	−8.13	0.03529	0.634	3.259	2.74	109	−40.9	−53.7 *−46.1*
O13	H23B ^ii^	2.3	−1.28	0.00743	0.122	4.084	3.37	124		
H13A	O14 ^ii^	7.86	−4.98	0.02567	0.394	3.613	2.84	128		
H14A	O23 ^ii^	12.93	−8.69	0.04038	0.63	3.429	2.63	130		
H14A	S24 ^ii^	6.97	−5.34	0.03615	0.316	4.137	3.07	167		
H23A	O14 ^iii^	22.51	−17.79	0.077	0.999	3.308	2.34	147	−11.2	−30.6 *−24.2*
H14B	O14 ^iii^	3.86	−2.43	0.01651	0.194					
H4	N5 ^iv^	19.54	−13.48	0.05478	0.94	3.413	2.48	143	−5.9	−27.1 *−22.7*

**Table 3:** The details of the interaction energies (in kJ/mol) for the four pairs from Table 2. Upper row: HF-3-21G; lower row, italics: B3LYP/6-31G(d,p). R is the distance between the centroids of the interacting molecules.

R	E_ele	E_pol	E_dis	E_rep	E_tot
5.13	−18.7 *−19.2*	−10.0 *−5.9*	−90.3 *−90.3*	46.7 *58.2*	−69.1 *−67.4*
7.84	−25.4 *−21.0*	−9.4 *−5.7*	−46.4 *−46.4*	24.7 *33.5*	−53.7 *−46.1*
8.78	−20.3 *−16.6*	−7.3 *−4.2*	−18.0 *−18.0*	13.6 *19.5*	−30.6 *−24.2*
11.39	−21.9 *−18.8*	−4.0 *−3.1*	−12.2 *−12.2*	10.9 *16.3*	−27.1 *−22.7*

**Table 4:** Details of the interactions for the chosen pairs of molecules in **4** (cf. Text). G_cp_: kinetic energy density (kJ/mol/Bohr^3^); V_cp_: potential energy density (kJ/mol/Bohr^3^); Lap: laplacian at the BCP (e·Å^−5^)]; Den: electron density at the BCP (e·Å^−3^), distances in Å, angles in °, and energies in kJ/mol. Symmetry codes: ^i^ ½ + x,½ − y,−z; ^ii^ ½ − x,1 − y,½ − z.

Atom1	Atom2	Gcp	Vcp	DEN	LAP	X···Y	H···Y	X-H···Y	pixel	HF
C2	C15 ^i^	4.51	−2.93	0.01966	0.224	4.382			−89.8	−66.4
C11	H26 ^i^	9.44	−6.67	0.03728	0.448	3.733	2.85	139		
O3	S14 ^i^	7.59	−5	0.02788	0.374	3.538				
H12	C16 ^i^	6.21	−3.89	0.02169	0.313	3.766	3.05	124		
H12	H15C ^i^	2.9	−1.98	0.01708	0.14	2.73				
H13C	H16 ^i^	11.03	−7.61	0.03883	0.531	2.28				
H13C	N5 ^i^	12.59	−10.2	0.05647	0.55	3.651	2.66	151		
H14B	C23 ^i^	7.24	−5.36	0.03481	0.335	3.953	2.98	150		
H15B	H24C ^i^	7.2	−4.85	0.02851	0.351	2.46				
C22	O15 ^i^	14.28	−10.14	0.04821	0.677	3.096				
H22	S14 ^i^	6.18	−4.27	0.02755	0.297	4.095	3.17	143		
H23	N5 ^ii^	56.52	−82.77	0.27482	1.112	2.763	1.83	156	−39.6	−60.0
O23	H15B ^ii^	12.04	−8.8	0.0461	0.561	3.620	2.60	155		
O23	H16 ^ii^	21.77	−15.63	0.06353	1.025	3.216	2.38	132		
O24	H4 ^ii^	15.58	−10.72	0.04758	0.75	3.211	2.63	113		

**Table 5:** Details of the interactions for the chosen pairs of molecules in **7** (cf. Text). G_cp_: kinetic energy density (kJ/mol/Bohr^3^); V_cp_: potential energy density (kJ/mol/Bohr^3^); Lap: laplacian at the BCP (e·Å^−5^)]; Den: electron density at the BCP (e·Å^−3^), distances in Å, angles in °, and energies in kJ/mol. Symmetry codes: ^i^ x,−1 + y,z; ^ii^ −x,−½ + y,−z.

Atom1	Atom2	Gcp	Vcp	DEN	LAP	X···Y	H···Y	X-H···Y	pixel	HF
C13	C15 ^i^	7.73	−5.48	0.03329	0.366	3.490			−105.0	−67.8
C4	N5 ^i^	2.08	−1.22	0.0089	0.108	4.083				
C16	H15B ^i^	11.62	−9.11	0.05108	0.519	3.673	2.69	150		
H12	H23B ^i^	7.18	−5.23	0.0336	0.335	2.29				
Br13	C231 ^i^	8.05	−5.3	0.02884	0.397	3.636				
Br13	O14 ^i^	9.33	−6.44	0.03524	0.449	3.505				
H14C	O14 ^i^	14.14	−11.04	0.05703	0.633	3.593	2.52	171		
H14C	O15 ^i^	9.03	−5.54	0.02533	0.459	3.463	2.87	115		
C22	H23B ^i^	8.91	−6.4	0.03717	0.419	3.900	2.89	155		
C24	S23 ^i^	7.59	−5.42	0.03341	0.358	3.674				
C25	C23 ^i^	6.72	−4.71	0.02986	0.321	3.600				
H24B	O24 ^i^	7.52	−5.33	0.03276	0.356	3.815	2.80	155		
Br13	Br13 ^ii^	12.28	−9.6	0.0526	0.549	3.564			−14.2	−14.4
Br13	H23A ^ii^	8.14	−5.2	0.02691	0.407	3.838	3.19	118		
O14	H23A ^ii^	4.73	−3.05	0.01997	0.235	4.057	3.00	165		
H14A	S23 ^ii^	3.24	−1.99	0.01374	0.165	4.232	3.51	125		

**Table 6:** Details of the interactions for the chosen pairs of molecules in **5** (cf. Text). G_cp_: kinetic energy density (kJ/mol/Bohr^3^); V_cp_: potential energy density (kJ/mol/Bohr^3^); Lap: laplacian at the BCP (e·Å^−5^); Den: electron density at the BCP (e·Å^−3^), distances in Å, angles in °, and energies in kJ/mol. Symmetry codes: ^i^ −x,2 − y,1 − z; ^ii^ 1 − x,1 − y,1 − z.

Atom1	Atom2	Gcp	Vcp	DEN	LAP	X···Y	H···Y	X-H···Y	pixel	HF
H14C	O3 ^i^	2.97	−1.78	0.01199	0.153	4.188	3.22	148	−76.0	−72.9
C15	H22 ^i^	6.48	−4.37	0.02686	0.315	3.803	2.99	131		
N5	C12 ^i^	6.97	−4.66	0.02749	0.341	3.568				
H14C	H22 ^i^	10.3	−7.12	0.03753	0.495	2.25				
C4	H13A ^i^	13.33	−9.18	0.04339	0.642	3.467	2.76	122		
C13	S14 ^ii^	4.69	−3.13	0.02168	0.229	3.886			−54.5	−54.0
H13C	S14 ^ii^	11.01	−8.54	0.04862	0.495	3.886	2.89	142		
H26	S14 ^ii^	11.53	−8.35	0.04412	0.54	3.693	2.86	133		

**Table 7:** Crystal data, data collection, and structure refinement.

Compound	1	2	3	4	5
Formula	C_20_H_21_NO_5_S	C_20_H_20_BrNO_5_S	C_19_H_19_NO_5_S	C_19_H_19_NO_5_S	C_19_H_18_FNO_4_S
Formula weight	387.44	466.34	373.41	373.41	375.40
Crystal system	triclinic	monoclinic	triclinic	orthorhombic	triclinic
Space group	P-1	P2_1_/c	P-1	P2_1_2_1_2_1_	P-1
a(Å)	5.1299(4)	12.3841(4)	7.7068(3)	8.3865(4)	7.8698(8)
b(Å)	11.4060(10)	7.7755(4)	10.2384(3)	11.1560(6)	10.4011(8)
c(Å)	16.0097(13)	21.2517(7)	11.9461(4)	19.1503(9)	12.0236(7)
α(°)	97.245(7)	90	108.544(3)	90	72.165(6)
β(°)	94.098(7)	101.623(3)	94.602(3)	90	88.285(6)
γ(°)	99.063(7)	90	100.912(3)	90	68.924(8)
V(Å^3^)	913.68(13)	2004.42(14)	867.47(5)	1791.70(15)	870.64(13)
Z	2	4	2	4	2
D_x_(g cm^−3^)	1.408	1.545	1.430	1.384	1.432
F(000)	408	952	392	784	392
μ(mm^−1^)	0.210	2.186	0.218	0.211	0.221
Reflections:					
collected	17518	19235	16633	6970	11038
unique (R_int_)	4187 (0.0778)	3463 (0.0708)	3837 (0.0227)	3324 (0.0439)	3493 (0.0246)
with I > 2σ(I)	2504	1493	3234	2876	3168
R(F) [I > 2σ(I)]	0.0620	0.0535	0.0369	0.0436	0.0325
wR(F^2^) [I > 2σ(I)]	0.1073	0.1045	0.0917	0.0986	0.0806
R(F) [all data]	0.1234	0.1402	0.0467	0.0594	0.0362
wR(F^2^) [all data]	0.1303	0.1117	0.0956	0.1052	0.0831
Goodness of fit	1.022	0.991	1.037	1.050	1.032
Flack parameter				−0.07(6)	
max/min Δ (e·Å^−3^)	0.45/−0.38	0.41/−0.53	0.48/−0.31	0.23/−0.26	0.27/−0.27
CCDC number	2040699	2040698	2040700	2040701	2040702
**Compound**	**6**	**7**	**8**	**9**	
Formula	C_20_H_21_NO_4_S	C_19_H_18_BrNO_4_S	C_19_H_18_BrNO_4_S	C_18_H_16_BrNO_4_S	
Formula weight	371.44	436.31	436.31	422.29	
Crystal system	triclinic	monoclinic	monoclinic	orthorhombic	
Space group	P-1	P2_1_/c	P2_1_/c	P2_1_2_1_2_1_	
a(Å)	8.97622(14)	12.4359(9)	19.8673(3)	8.2803(3)	
b(Å)	9.83530(14)	4.1836(3)	4.79320(10)	11.3223(2)	
c(Å)	21.8875(3)	17.9998(12)	20.3846(4)	19.3117(4)	
α(°)	101.0102(13)	90	90	90	
β(°)	94.2346(13)	103.988(7)	105.164(2)	90	
γ(°)	90.0267(12)	90	90	90	
V(Å^3^)	1891.36(5)	908.70(11)	1873.59(6)	1810.51(8)	
Z	4	2	4	4	
D_x_(g cm^−3^)	1.304	1.595	1.547	1.549	
F(000)	784	444	888	856	
μ(mm^−1^)	1.728	2.402	4.244	2.408	
Reflections:					
collected	36875	9380	16185	10218	
unique (R_int_)	36875 (0.0160)	3151 (0.0907)	3535 (0.0291)	3269 (0.0307)	
with I > 2σ(I)	32098	1829	3067	2882	
R(F) [I > 2σ(I)]	0.0656	0.0593	0.0392	0.0336	
wR(F^2^) [I > 2σ(I)]	0.1879	0.0605	0.1147	0.0755	
R(F) [all data]	0.0722	0.1341	0.0479	0.0415	
wR(F^2^) [all data]	0.1879	0.0703	0.1206	0.0782	
Goodness of fit	1.024	0.965	1.045	1.045	
Flack parameter				−0.002(4)	
max/min Δ (e·Å^−3^)	0.57/−0.32	0.40/−0.32	0.89/−0.82	0.66/−0.37	
CCDC number	2040703	2040704	2040705	2040706	

## Data Availability

Crystallographic data for the structural analysis has been deposited with the Cambridge Crystallographic Data Centre. Copies of this information may be obtained free of charge from: The Director, CCDC, 12 Union Road, Cambridge, CB2 1EZ. UK; e-mail: deposit@ccdc.cam.ac.uk or www: www.ccdc.cam.ac.uk (accessed on 1 May 2021).

## Supplementary Materials

The following are available online: Figures S1–S6: perspective views of molecules **2**, **3**, **4**, **6**, **7**, and **8**; Figure S7: a comparison of the crystal packings of isostructural pairs **4**–**9**; Tables S1–S8: interaction data for compounds **1**–**7** and **9**.

## Sample Availability

Samples of the compounds **1**–**9** are available from the authors.
