# Supplementary material for: Weak Intermolecular Interactions in a Series of Bioactive Oxazoles
Source: Molecules. 2021 May 19;26(10):3024. doi: 10.3390/molecules26103024 (PMC8159086; doi:10.3390/molecules26103024)
Supplement: Supplementary file 1 [file molecules-26-03024-s001.zip › molecules-1204518-SI.pdf]

Supplementary information for a paper:

Weak intermolecular interactions in a series of bioactive oxazoles

Anita M. Grześkiewicz, Tomasz Stefański and Maciej Kubicki[1]*

[1] Faculty of Chemistry, Adam Mickiewicz University, Uniwersytetu Poznanskiego 8, 61-614 Poznań, Poland

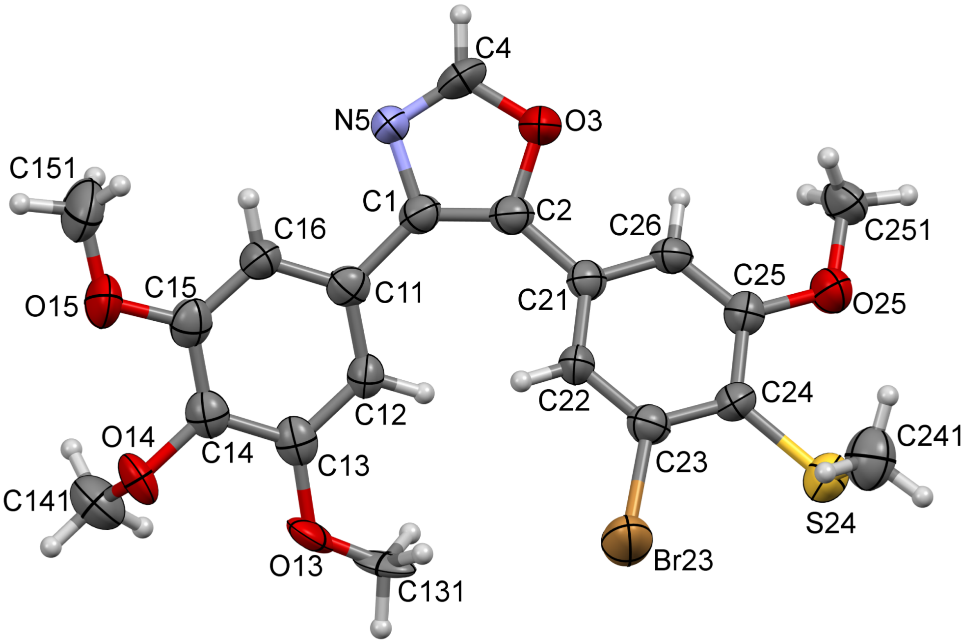

Figure 1. A perspective view of the molecule **2**; ellipsods are dran at the 50% probability level, hydrogen atoms are shown as spheres of arbitrary radii.

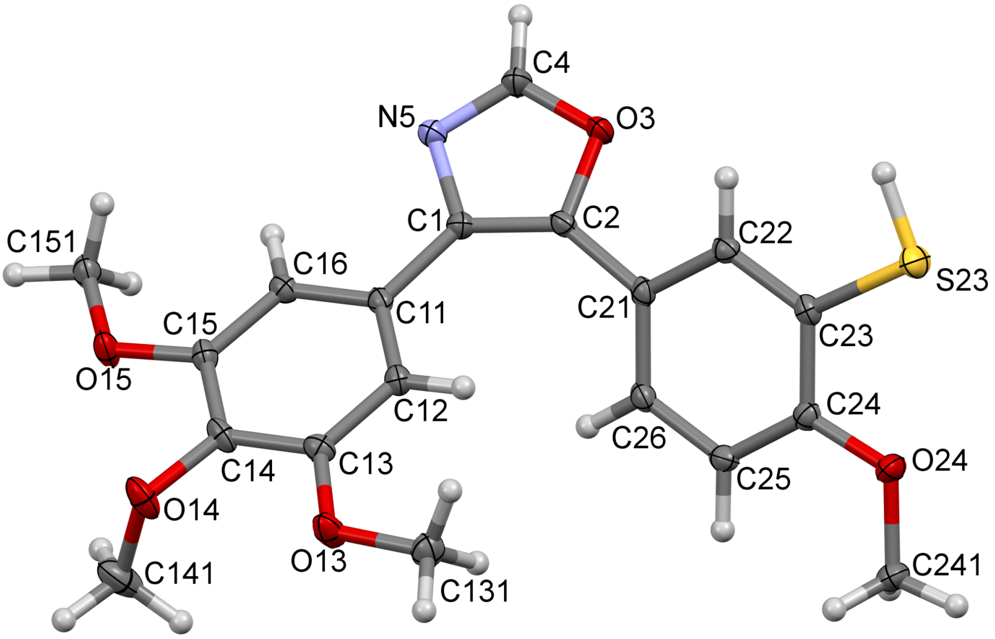

Figure 2. A perspective view of the molecule **3**; ellipsods are dran at the 50% probability level, hydrogen atoms are shown as spheres of arbitrary radii.

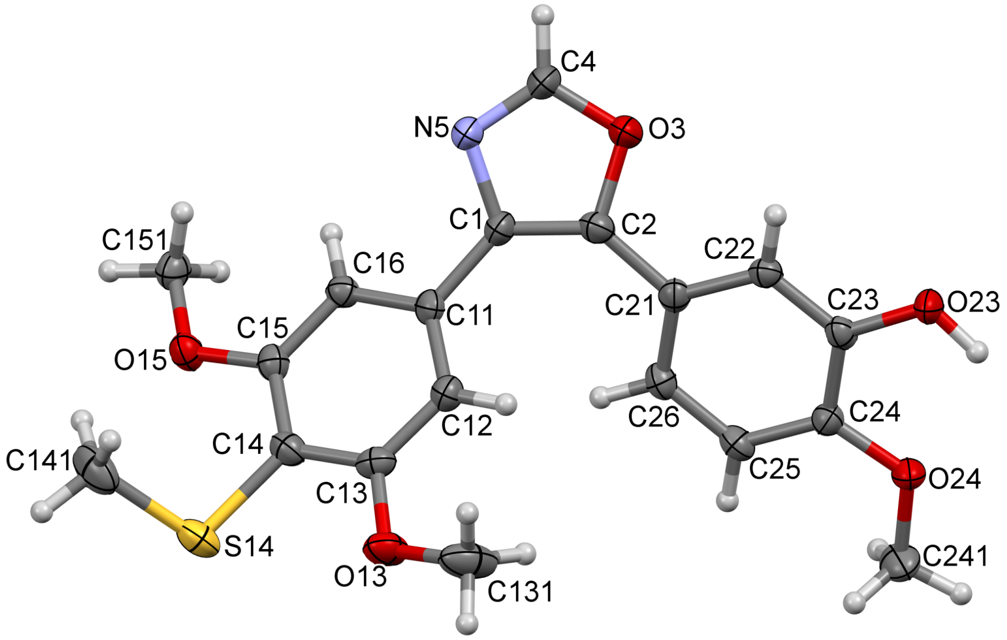

Figure 3. A perspective view of the molecule **4**; ellipsods are dran at the 50% probability level, hydrogen atoms are shown as spheres of arbitrary radii.

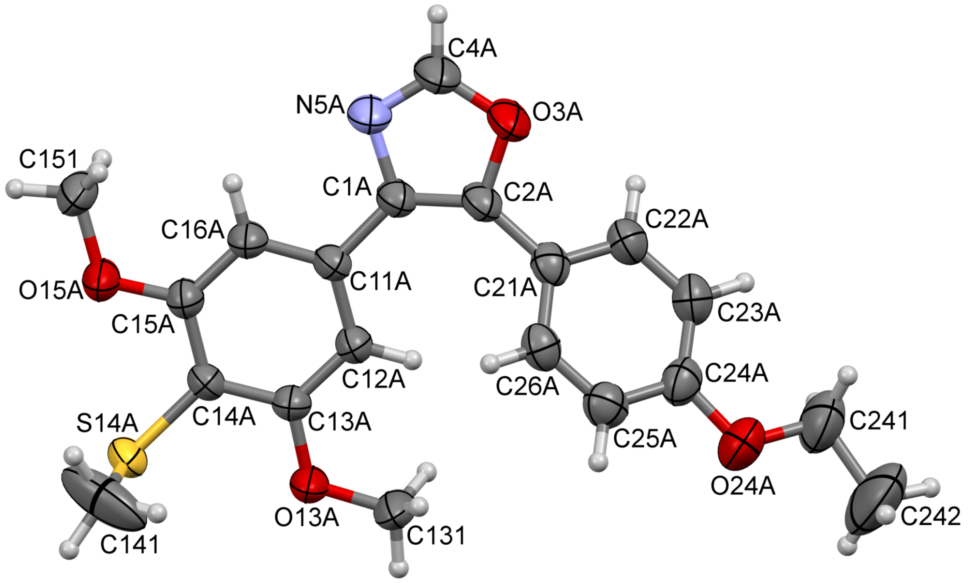

Figure 4. A perspective view of the molecule **6A**; ellipsods are dran at the 50% probability level, hydrogen atoms are shown as spheres of arbitrary radii.

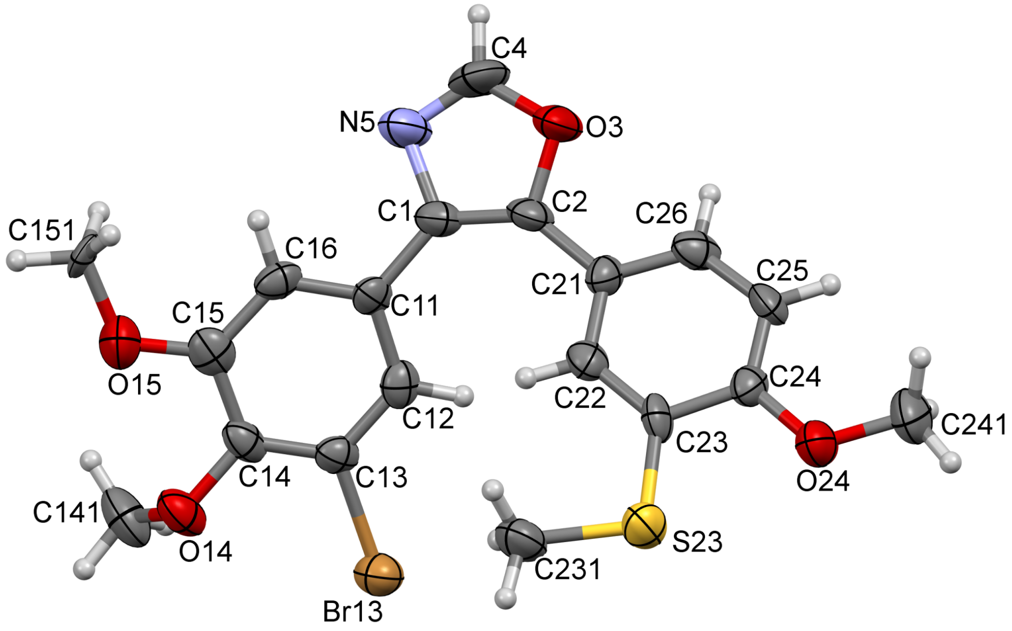

Figure 5. A perspective view of the molecule **7**; ellipsods are dran at the 50% probability level, hydrogen atoms are shown as spheres of arbitrary radii.

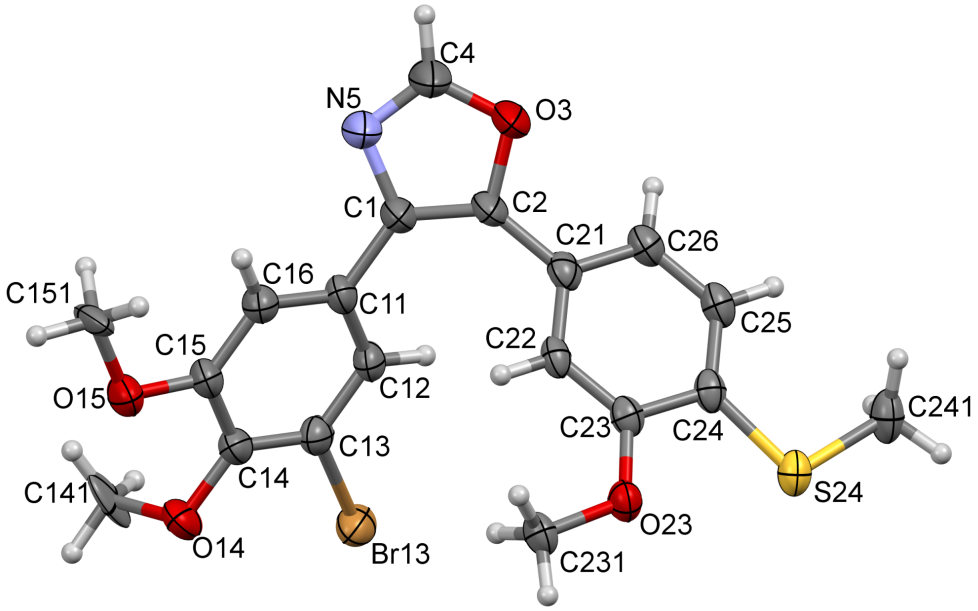

Figure 6. A perspective view of the molecule **9**; ellipsods are dran at the 50% probability level, hydrogen atoms are shown as spheres of arbitrary radii.

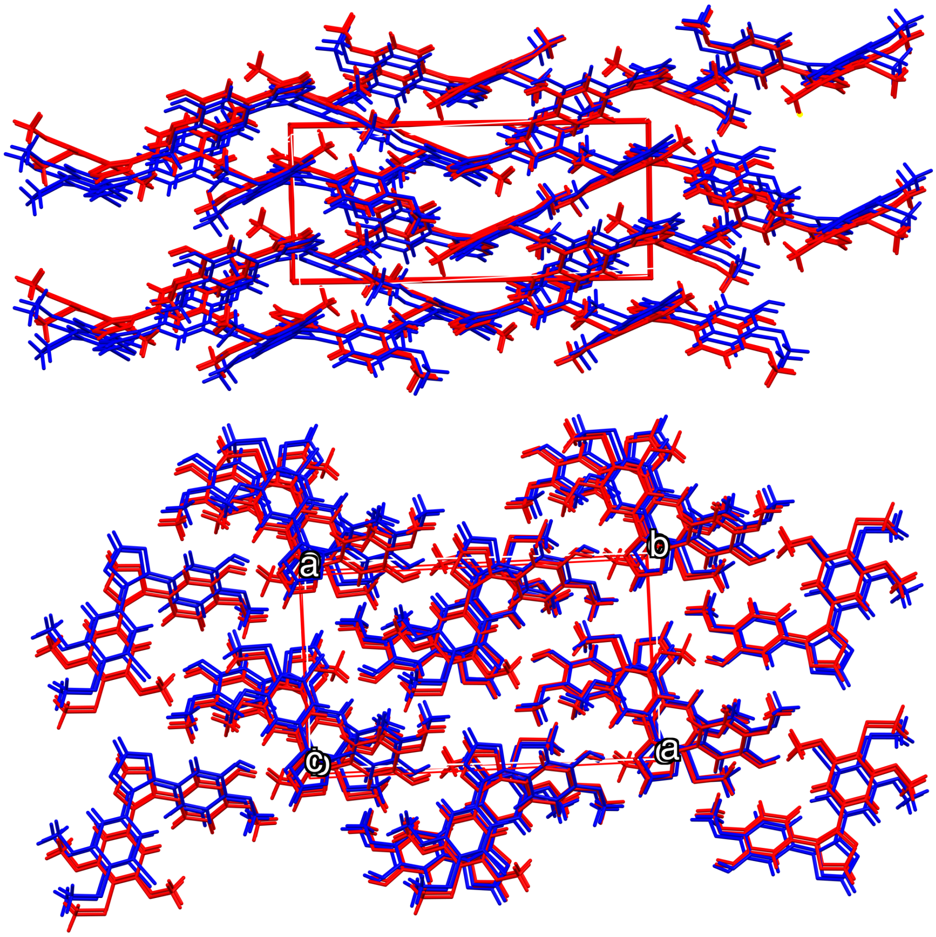

Fig. 7. Two comparisons of crystal packings of compounds **4** and **9**, showing high degree of their isostructurality

Tables 1 – 8. The details of the interactions for the pairs of molecules for which critical points were found. G_{cp}: kinetic energy density (kJ/mol/Bohr^3); V_{cp}: potential energy density (kJ/mol/Bohr^3); DEN: electron density at the BCP (e·Å^{-3}), LAP: laplacian at the BCP (e·Å^{-5}); distances in Å, angles in °, energies in kJ/mol.

Table 1: 1

Atom1	Atom2	sym2	Gcp	Vcp	DEN	LAP	X···Y	H···Y	D-H···Y	Pix	HF
C16	C12	45501	4,35	-2,77	0,01834	0,218	3,742			-78.9	-69.1
C14	H13C	45501	8,55	-6,18	0,03675	0,401	3.717	2.90	131		
O15	H14B	45501	10,27	-6,41	0,02888	0,519	3.447	2.78	119		
O14	H14B	45501	3,86	-2,43	0,01651	0,194	4.173	3.14	158		
H15C	C11	45501	8,86	-7,02	0,0442	0,393	3.980	2.90	170		
H22	H12	45501	0,98	-0,58	0,00581	0,051	3.39				
O23	H24A	45501	14,83	-10,65	0,05047	0,698	3.452	2.53	142		
H23C	C24	45501	12,94	-10,26	0,05547	0,574	3.550	2.69	136		
S24	H24A	45501	9,07	-6,53	0,03773	0,427	3.894	2.96	144		
H16	H24B	46501	3,11	-1,98	0,01485	0,156	2,70			-8.8	-10.0
C151	S24	46501	6,26	-3,9	0,02138	0,316	3.707				
O23	H15B	54501	11,62	-7,96	0,03949	0,561	3.504	2.65	135	-21.9	-18.0
H23A	H15A	54501	5,94	-3,82	0,02275	0,295	2.50				
S24	H15B	54501	8,29	-6,68	0,04365	0,363	4.022	2.97	164		
S24	C16	54501	6,38	-4,33	0,02705	0,309	3.736				
H24B	N5	54501	12,44	-8,19	0,03749	0,613	3.406	2.73	120		
H23A	O14	56602	22,51	-17,79	0,077	0,999	3.308	2.34	147	-11.2	-30.6
H14B	O14	56602	3,86	-2,43	0,01651	0,194					
H15A	C141	57602	8,09	-5,61	0,03267	0,388	3.916	2.87	160	-10.0	-14.5
H13A	O13	66602	12,71	-8,13	0,03529	0,634	3.259	2.74	109	-40.9	-53.7
O13	H23B	66602	2,3	-1,28	0,00743	0,122	4.084	3.37	124		
H13A	O14	66602	7,86	-4,98	0,02567	0,394	3.613	2.84	128		
H14A	O23	66602	12,93	-8,69	0,04038	0,63	3.429	2.63	130		
H14A	S24	66602	6,97	-5,34	0,03615	0,316	4.137	3.07	167		
O3	C24	66702	5,62	-3,55	0,02087	0,282	3.590			-35.3	-25.1
H4	S24	66702	9	-6,27	0,03517	0,43	3.790	3.13	120		
C26	C26	66702	1,39	-0,81	0,00665	0,073	4.440				
H24C	C4	66702	11,76	-8,07	0,03994	0,567	3.602	2.79	131		
H14C	O15	67602	12,69	-8,23	0,03649	0,629	3.376	2.67	122	-12.7	-15.4

H14C	H14C	67602	12,35	-10,16	0,05718	0,534	2.09				
H4	N5	67702	19,54	-13,48	0,05478	0,94	2.48	3.413	143	-5.9	-27.1
H13A	H13A	76602	13,55	-9,05	0,04088	0,663	2.21			-4.3	-6.1
O3	H24C	76702	9,12	-5,71	0,02712	0,46	3.423	2.88	111	-15.4	-19.7
O3	H25	76702	3,98	-2,5	0,01664	0,201	4.166	3.08	173		
H26	H25	76702	15,29	-10,96	0,05126	0,72	2.11				

Table 2: 2

Atom1	Atom2	sym2	Gcp	Vcp	DEN	LAP	X···Y	H···Y	D-H···Y	Pix	HF
H15A	O25	45404	10,74	-7,88	0,04334	0,499	3.689	2.64	162	-13.3	-17.6
H4	Br23	55404	19,63	-15,18	0,0684	0,884	3.466	2.77	121	-10.2	-17.3
H4	S24	55404	15,23	-10,21	0,04421	0,743	3.844	2.78	166		
H13A	O14	64502	15,02	-9,85	0,04152	0,742	3.183	2.68	108	-25.1	-20.4
N5	H24C	74502	10,54	-7,02	0,0349	0,516	3.578	2.74	133	-108.9	-86.9
O13	H25C	74502	8,81	-6,64	0,04043	0,403	3.897	2.81	173		
H13B	O3	74502	12,71	-9,11	0,04581	0,599	3,541	2.58	147		
C16	H24B	74502	7,65	-5,17	0,02985	0,372	3.732	2.87	136		

Table 3: 3

Atom1	Atom2	sym2	Gcp	Vcp	DEN	LAP	X···Y	H···Y	D-H···Y	Pix	HF
H23	H15A	44401	8,05	-5,33	0,0293	0,395	2.51			-8.8	-4.8
S23	H14B	44401	7,75	-6	0,03922	0,349	4.067	3.01	164		
H24C	H14A	54401	5,56	-3,62	0,02241	0,275	2.59			-10.3	5.0
O3	H13A	54501	6,79	-4,25	0,02264	0,343	3.703	2.89	132	-10.0	-24.0
H4	H14C	54501	13,28	-8,66	0,038	0,658	2.24				
H4	O13	54501	23,78	-16,67	0,0638	1,134	3.297	2.35	144		
H24B	H15B	55401	14,22	-11,26	0,05858	0,631	2.20			-6.8	-13.1
H24A	H16	55401	8,27	-4,98	0,02258	0,424	2.56				
H24A	N5	55401	13,25	-9,8	0,04993	0,613	3.560	2.63	143		
H22	S23	55502	6,42	-4,12	0,0235	0,321	3.788	3.28	110	-18.4	-17.5
S23	S23	55502	6,96	-4,72	0,02843	0,338	3.859				
C131	S23	56502	8,68	-5,7	0,03004	0,428	3.654			-39.5	-55.7
H13C	H24A	56502	12,39	-8,91	0,04547	0,583	2.18				
C23	H24B	56502	15,1	-12,57	0,06568	0,647	3.619	2.63	151		
C1	C12	56602	5,61	-3,74	0,02398	0,274	3.641			-85.6	-87.4
N5	H13C	56602	12,8	-9,47	0,04889	0,592	3.691	2.71	150		

C15	H22	56602	9,11	-6,17	0,03331	0,442	3.599	2.88	123		
O15	H23	56602	7,92	-5,28	0,02946	0,388	3.728	2.85	129		
C151	S23	56602	6,77	-4,32	0,02392	0,339	3.714				
O13	O13	57602	9,53	-6	0,02832	0,48	3.209			-18.5	-32.1
H13A	O14	57602	12,37	-8,14	0,03734	0,609	3.430	2.65	128		
H24C	C25	66502	6,68	-4,87	0,03224	0,312	4.037	2.97	167	-11.3	-12.1
C1	C1	66602	5,69	-3,81	0,02433	0,278	3.616			-74.4	-66.8
C2	C16	66602	7,49	-5,1	0,02998	0,363	3.494				
N5	H26	66602	8,53	-5,72	0,03132	0,416	3.708	2.80	141		
H16	C26	66602	7,67	-5,32	0,03159	0,368	3.815	2.99	146		
H15C	C21	66602	14,53	-12,81	0,06975	0,596	3.483	2.52	147		
H13B	C141	67602	9,35	-6,38	0,03435	0,452	3.843	2.88	148	-21.1	-20.7
H25	H14A	67602	7,25	-4,44	0,02209	0,369	2.44				
H26	H14A	67602	5,7	-3,28	0,01504	0,298	2.63				
H15A	O15	67702	19,69	-15,4	0,06987	0,88	3.388	2.40	151	-9.7	-26.2

Table 4: 4

Atom1	Atom2	sym2	Gcp	Vcp	DEN	LAP	X···Y	H···Y	D-H···Y	Pix	HF
C2	C15	46703	4,51	-2,93	0,01966	0,224	4.382			-89.8	-66.4
C11	H26	46703	9,44	-6,67	0,03728	0,448	3.733	2.85	139		
O3	S14	46703	7,59	-5	0,02788	0,374	3.538				
H12	C16	46703	6,21	-3,89	0,02169	0,313	3.766	3.05	124		
H12	H15C	46703	2,9	-1,98	0,01708	0,14	2.73				
H13C	H16	46703	11,03	-7,61	0,03883	0,531	2.28				
H13C	N5	46703	12,59	-10,2	0,05647	0,55	3.651	2.66	151		
H14B	C23	46703	7,24	-5,36	0,03481	0,335	3.953	2.98	150		
H15B	H24C	46703	7,2	-4,85	0,02851	0,351	2.46				
C22	O15	46703	14,28	-10,14	0,04821	0,677	3.096				
H22	S14	46703	6,18	-4,27	0,02755	0,297	4.095	3.17	143		
H4	S14	54501	7,33	-4,8	0,027	0,362	3.949	3.14	132	-8.3	-12.8
H23	N5	66402	56,52	-82,77	0,27482	1,112	2.763	1.83	156	-39.6	-60.0
O23	H15B	66402	12,04	-8,8	0,0461	0,561	3.620	2.60	155		
O23	H16	66402	21,77	-15,63	0,06353	1,025	3.216	2.38	132		
O24	H4	66402	15,58	-10,72	0,04758	0,75	3.211	2.63	113		
H13B	H14C	67402	2,5	-1,41	0,0081	0,132	2.89			-10.6	-12.2

H13A	H15A	67402	4,66	-2,88	0,01743	0,236	2.60				
C24	H14A	67402	10,8	-7,83	0,04251	0,505	3.631	2.72	141		
H24B	H14B	67402	17,2	-13,15	0,06198	0,78	2.04				
O3	H24A	74604	7,96	-4,78	0,02182	0,409	3.500	2.89	116	-20.6	-34.2
C22	H24B	74604	13,47	-9,77	0,04857	0,63	3.422	2.66	127		
O23	H25	74604	12,77	-9,4	0,04835	0,593	3.590	2.55	160		
H24C	H13A	74604	16,42	-10,95	0,04572	0,804	2.23				
H24A	O13	74604	11,5	-7,38	0,0335	0,573	3.467	2.71	126		
C151	H14A	74704	16,71	-11,64	0,05089	0,8	3.421	2.66	127	-2.9	-7.4

Table 5: 5

Atom1	Atom2	sym2	Gcp	Vcp	DEN	LAP	X···Y	H···Y	D-H···Y	Pix	HF
F23	H15B	46401	18,96	-13,03	0,05336	0,914	3.203	2.38	131	-2.7	-2.5
H13B	O3	54501	5,68	-3,58	0,02079	0,286	3.801	2.92	138	-9.8	-28.2
O13	H4	54501	18,27	-12,32	0,05001	0,889	3.398	2.48	142		
S14	H4	54501	19,03	-13,93	0,06082	0,886	3.637	2.70	144		
H14B	O24	54601	21	-16,63	0,07406	0,932	3.377	2.38	152	-8.1	-8.9
H24B	H16	55401	7,96	-4,83	0,02262	0,407	2.50			-7.4	-10.9
H24B	N5	55401	10,61	-7,54	0,04046	0,502	3.657	2.73	143		
H24C	H15C	55401	11,67	-8,78	0,04767	0,535	2.26				
H13B	H14C	56602	8,12	-5,38	0,02948	0,399	2.34			-13.9	-9.9
H13B	F23	57502	2,42	-1,31	0,00626	0,13	3.837	3.32	110	-31.2	-45.9
H13A	H24B	57502	5,88	-3,72	0,02159	0,295	2.57				
H13A	O24	57502	7,34	-4,4	0,02066	0,378	3.504	2.93	113		
C23	H24C	57502	15,65	-11,32	0,05288	0,734	3.371	2.63	124		
H14C	O3	57602	2,97	-1,78	0,01199	0,153	4.188	3.22	148	-76.0	-72.9
C15	H22	57602	6,48	-4,37	0,02686	0,315	3.803	2.99	131		
N5	C12	57602	6,97	-4,66	0,02749	0,341	3.568				
H14C	H22	57602	10,3	-7,12	0,03753	0,495	2.25				
C4	H13A	57602	13,33	-9,18	0,04339	0,642	3.467	2.76	122		
C13	S14	66602	4,69	-3,13	0,02168	0,229	3.886			-54.5	-54.0
H13C	S14	66602	11,01	-8,54	0,04862	0,495	3.886	2.89	142		
H26	S14	66602	11,53	-8,35	0,04412	0,54	3.693	2.86	133		
H15B	H15B	66702	5,99	-3,46	0,0157	0,313	2.78			-6.9	-11.0
H15B	H14A	66702	8,86	-6,2	0,03512	0,423	2.27				

H24A	C25	67502	8,86	-7,05	0,04446	0,392	3.895	2.81	175	-13.8	-14.0
N5	H26	67602	4,69	-2,89	0,01739	0,238	3.920	3.07	135	-64.6	-55.3
C1	N5	67602	5,45	-3,56	0,02242	0,269	3.648				
H15A	C21	67602	8,34	-6,18	0,03798	0,385	3.846	2.90	145		
O3	C16	67602	8,97	-6,05	0,03267	0,436	3.345				
O3	H15A	67602	10,96	-8,04	0,04391	0,509	3.655	2.64	155		

Table 6: 6

Atom1	Atom2	sym2	Gcp	Vcp	DEN	LAP	X···Y	H···Y	D-H···Y	Pix	HF
H13C	C4A	45501	4,2	-2,67	0,01778	0,21	3.988	3.22	128	-13.9	-5.8
H25A	O3A	45501	0,45	-0,24	0,00208	0,024	5.164	4.19	155		
H14A	N5A	45501	1,26	-0,71	0,00562	0,066	4.637	3.66	150		
H14B	H15C	45501	5,12	-3,3	0,02086	0,255	2.50				
H14D	N5B	45501	1,26	-0,72	0,0059	0,066	4.732	3.69	160	-14.4	-9.1
H14D	H16B	45501	1,85	-1,07	0,00786	0,097	3.00				
H14E	H15F	45501	9,55	-6,02	0,02851	0,48	2.34				
H13F	C4B	45501	9,74	-6,67	0,03549	0,47	3.490	2.86	117		
H13E	O3A	45501	5,78	-3,59	0,02015	0,292	3.780	2.94	134	-12.2	-27.7
S14B	H4A	45501	14,93	-10,65	0,05001	0,705	3.753	2.80	146		
O13B	H4A	45501	22,27	-15,52	0,06057	1,066	3.328	2.40	143		
H24I	H24E	54501	2,77	-1,64	0,01094	0,143	2.82			-93.6	-73.2
H24G	H24B	54501	3,31	-1,92	0,01126	0,173	2.92				
H24F	O24A	54501	4,44	-2,83	0,01849	0,222	3.964	3.11	135		
C25B	H13A	54501	6,16	-4,7	0,03327	0,28	4.088	3.01	173		
H25B	C23A	54501	7,78	-5,74	0,03618	0,36	3.917	2.89	158		
S14A	C142	56501	3,24	-1,9	0,01159	0,168	4.156				
H15C	N5B	56501	7,28	-4,99	0,02986	0,352	3.822	2.87	146		
C2A	H26B	56501	9	-6,67	0,03975	0,416	3.834	2.85	151		
S14A	C12B	56501	9,49	-7,56	0,04638	0,42	3.510				
C12A	C26B	56501	10,51	-7,42	0,03963	0,499	3.358				
O13A	H13F	56501	14,58	-11,27	0,05718	0,657	3.572	2.52	163		
H15A	H15E	55501	3,7	-2,42	0,01779	0,183	2.71			-81.8	-61.2
H14A	S14B	55501	5,99	-4,04	0,02568	0,291	4.005	3.16	135		
C25A	H13D	55501	6,59	-4,26	0,02447	0,327	3.881	3.04	134		

C13A	S14B	55501	6,65	-4,79	0,03143	0,312	3.735				
C16A	O15B	55501	7,93	-5,21	0,02847	0,391	3.358				
C26A	H22B	55501	8,54	-6,53	0,04071	0,387	3.873	2.85	156		
O24A	H23B	55501	10,22	-7,35	0,04049	0,48	3.781	2.74	161		
H15F	N5A	55501	10,59	-7,7	0,0422	0,495	3.647	2.70	145		
H26A	C13B	55501	14,77	-10,8	0,05209	0,688	3.414	2.62	129		
O3B	H13B	64501	10,62	-7,18	0,03631	0,516	3.549	2.67	137	-12.4	-26.3
H4B	S14A	64501	13,45	-9,7	0,04802	0,631	3.756	2.86	140		
H4B	O13A	64501	24,8	-18,08	0,07072	1,158	3.232	2.37	134		
H24H	H24F	64602	10,44	-7,78	0,04391	0,481	2.18			-5.2	-6.5
H25A	C242	65602	1,57	-0,88	0,00626	0,083	4.424	3.66	129	-9.5	-8.4
O24A	C242	65602	2,21	-1,27	0,00843	0,116	4.018				
H24E	O24B	65602	2,62	-1,58	0,01141	0,134	4.267	3.27	153	-10.9	-11.1
H24E	C23B	65602	3,04	-1,8	0,01162	0,157	4.207	3.37	135		
H13E	H24D	65602	11,71	-7,82	0,03741	0,573	2.26				
H24I	H13B	65602	13,34	-8,67	0,03779	0,662	2.24				
H14E	H14E	66702	1,87	-1,03	0,00598	0,1	3.00			-3.1	-2.1
H14B	C141	67702	1,19	-0,65	0,00429	0,064	3.764	3.53	96	-4.1	-2.6
H24G	C25B	74602	9,8	-7,75	0,04678	0,435	3.861	2.79	158	-15.1	-16.7
H24A	C23A	75602	5,06	-3,51	0,02468	0,243	4.176	3.11	168	-14.1	-13.9
H24D	C2B	75602	2,1	-1,25	0,00944	0,108	4.449	3.55	141	-24.6	-29.3
H24B	C26B	75602	5,22	-3,67	0,02588	0,248	4.156	3.15	154		
H23A	C24B	75602	9,63	-7,37	0,04379	0,437	3.685	2.77	142		
H24J	H22A	75602	12,28	-8,95	0,04643	0,573	2.12				
H15A	H14F	76702	7,06	-4,53	0,02498	0,352	2.44			-19.3	-27.7
H14C	H15D	76702	9,39	-6,91	0,0402	0,436	2.21				
H15A	O15B	76702	9,66	-6,12	0,02908	0,485	3.531	2.80	125		
H15D	O15A	76702	10,12	-6,73	0,03393	0,496	3.569	2.72	135		
H15E	S14B	76702	8	-6,08	0,03876	0,364	4.913	3.06	147	-13.3	-20.0
H15E	O15B	76702	16,73	-12,37	0,05742	0,774	3.412	2.48	143		
H15B	S14A	77702	9,53	-7,28	0,04337	0,433	3.914	2.98	144	-13.5	-11.1

H15B	O15A	77702	16,7	-12	0,05429	0,785	3.371	2.50	137		

Table 7: 7

Atom1	Atom2	sym2	Gcp	Vcp	DEN	LAP	X···Y	H···Y	D-H···Y	Pix	HF
S23	H15A	44501	2,67	-1,63	0,01198	0,136	4.433	3.56	139	-8.3	-13.1
S23	C151	45501	10,63	-7	0,03409	0,524	3.618			-13.5	-20.8
H24A	N5	45501	6,96	-4,76	0,02893	0,336	3.846	2.88	148		
O24	H15C	45501	6,49	-4,44	0,02768	0,314	3.943	2.87	167		
C13	C15	54501	7,73	-5,48	0,03329	0,366	3.490			-105.0	-67.8
C4	N5	54501	2,08	-1,22	0,0089	0,108	4.083				
C16	H15B	54501	11,62	-9,11	0,05108	0,519	3.673	2.69	150		
H12	H23B	54501	7,18	-5,23	0,0336	0,335	2.29				
Br13	C231	54501	8,05	-5,3	0,02884	0,397	3.636				
Br13	O14	54501	9,33	-6,44	0,03524	0,449	3.505				
H14C	O14	54501	14,14	-11,04	0,05703	0,633	3.593	2.52	171		
H14C	O15	54501	9,03	-5,54	0,02533	0,459	3.463	2.87	115		
C22	H23B	54501	8,91	-6,4	0,03717	0,419	3.900	2.89	155		
C24	S23	54501	7,59	-5,42	0,03341	0,358	3.674				
C25	C23	54501	6,72	-4,71	0,02986	0,321	3.600				
H24B	O24	54501	7,52	-5,33	0,03276	0,356	3.815	2.80	155		
Br13	Br13	54502	12,28	-9,6	0,0526	0,549	3.564			-14.2	-14.4
Br13	H23A	54502	8,14	-5,2	0,02691	0,407	3.838	3.19	118		
O14	H23A	54502	4,73	-3,05	0,01997	0,235	4.057	3.00	165		
H14A	S23	54502	3,24	-1,99	0,01374	0,165	4.232	3.51	125		
O3	H24C	54602	9,61	-6,83	0,03804	0,455	3.667	2.67	152	-22.5	-20.3
H26	C25	54602	8,09	-5,55	0,03193	0,39	3.745	2.86	138		
H25	C26	54602	8,04	-5,6	0,03278	0,385	3.750	2.84	141		
C241	O3	54602	10,93	-7,09	0,03334	0,542	3.323				
H26	H24C	54602	4,22	-2,92	0,02203	0,202	2.57				
H15A	H14A	65502	8,67	-6,53	0,04007	0,396	2.27			-6.2	-7.9
C4	H4	65602	10,8	-7,47	0,03865	0,519	3.601	2.87	110	-8.0	-12.8

Table 8: 9

Atom1	Atom2	sym2	Gcp	Vcp	DEN	LAP	X···Y	H···Y	D-H···Y	Pix	HF
C2	C15	45503	0,00144	-0,00092	0,01679	0,189	3.894			-92.4	-67.3
C12	H26	45503	0,00385	-0,00279	0,04078	0,473	3.728	2.85	138		
O3	S14	45503	0,00224	-0,00143	0,022	0,294	3.667				
H12	C16	45503	0,00325	-0,00216	0,03051	0,419	3.645	2.87	128		
H12	H15C	45503	0,00115	-0,00076	0,01623	0,149	2.75				
Br13	C1	45503	0,00294	-0,00205	0,03203	0,37	3.712				
H14A	C24	45503	0,00414	-0,00321	0,04813	0,489	3.760	2.78	149		
C151	H24C	45503	0,00236	-0,00154	0,02422	0,306	4.083	3.06	158		
H22	S14	45503	0,00272	-0,00187	0,02954	0,345	4,000	3.13	137		
C22	O15	45503	0,00517	-0,00366	0,04649	0,644	3.103				
C23	C151	45503	0,00417	-0,00288	0,03876	0,527	3.381				
H4	Br13	56501	0,00113	-0,00066	0,01093	0,154	4.352	3.66	123	-8.7	-11.7
H4	S14	56501	0,00289	-0,00185	0,026	0,378	3.997	3.09	141	-10.6	-13.4
H14B	C24	65402	0,00497	-0,00389	0,05456	0,584	3.683	2.62	166		
C141	H24A	65402	0,00528	-0,00371	0,04628	0,661	3.564	2.76	130		
H14C	Br13	65402	0,00015	-0,00008	0,00183	0,021	5.341	4.50	136		
H15B	Br13	65402	0,00026	-0,00014	0,00327	0,037	5.107	4.26	137		
N5	H23	66402	0,01969	-0,02646	0,24045	1,245	2.791	1.89	150	-39.6	-46.5
H4	O24	66402	0,00493	-0,0033	0,03985	0,633	3.266	2.72	111		
H16	O23	66402	0,00834	-0,00595	0,06301	1,033	3.198	2.39	130		
H15A	O23	66402	0,00541	-0,00408	0,05395	0,65	3.560	2.53	157		
H14B	C151	74404	0,00438	-0,00278	0,03245	0,577	3.449	2.92	110	-6.1	-7.5
Br13	H24B	74504	0,00478	-0,00327	0,0414	0,605	3.612	2.98	117	-18.8	-27.0
H25	O23	74504	0,00328	-0,00231	0,03487	0,41	3.770	2.71	164		
H24B	O3	74504	0,00367	-0,00228	0,02746	0,488	3.474	2.78	122		
H24A	C22	74504	0,00525	-0,00385	0,05026	0,641	3.422	2.64	128		
